# From Prenatal Intention to Postpartum Practice: A Prospective Cohort Study of Oral Health Preventive Behaviours in Mother–Child Pairs

**DOI:** 10.3390/healthcare14213652

**Published:** 2026-10-28

**Authors:** Cristina Ioana Bica, Oana Elena Stoica, Alexandra Mihaela Stoica, Liana Beresescu, Daniela Esian

**Affiliations:** 1 Department of Pedodontics, “George Emil Palade” University of Medicine, Pharmacy, Science, and Technology of Târgu Mureș, 540139 Târgu Mureș, Romania; cristina.bica@umfst.ro (C.I.B.); oana.stoica@umfst.ro (O.E.S.); daniela.esian@umfst.ro (D.E.); 2 Department of Odontology and Oral Pathology, “George Emil Palade” University of Medicine, Pharmacy, Science, and Technology of Târgu Mureș, 540139 Târgu Mureș, Romania; alexandra.stoica@umfst.ro; 3 Department of Preventive and Community Dentistry, “George Emil Palade” University of Medicine, Pharmacy, Science, and Technology of Târgu Mureș, 540139 Târgu Mureș, Romania

**Keywords:** early childhood caries, preventive behaviours, intention, pregnancy, infant oral health, cohort study

## Abstract

Background/Objectives: Preventive intentions formed during pregnancy do not always translate into practice after birth. We assessed how far prenatal intentions for the child’s oral health were reflected in behaviours at 12 months, and which maternal factors were associated with them. Methods: In this prospective cohort, 218 women in the third trimester reported their knowledge and intentions for six preventive behaviours and underwent an oral examination (International Caries Detection and Assessment System, ICDAS; Plaque Control Record, PCR). At 12 months postpartum, mothers reported the behaviours adopted. Data were analysed with the exact McNemar test and multivariable logistic regression. Results: Complete data were available for 159 pairs. At 12 months, favourable behaviours were significantly less frequent than favourable intentions in all six domains (*p* < 0.05), with the largest intention–behaviour gaps for twice-daily toothbrushing (83.0% vs. 38.4%) and avoidance of added sugar (86.8% vs. 40.3%). Only 10.1% of children had seen a dentist by 12 months. Prenatal intention was independently associated with toothbrushing from the first tooth (aOR 3.18; 95% CI 1.16–8.69), twice-daily toothbrushing (aOR 2.51; 95% CI 1.02–6.17), fluoride toothpaste use (aOR 36.20; 95% CI 12.40–105.70) and avoidance of night-time bottles (aOR 6.29; 95% CI 3.08–12.82), but not with avoidance of added sugar; after correction for multiple comparisons, only the associations for fluoride toothpaste and night-time bottles remained significant. Exploratory analyses suggested that higher maternal PCR may be associated with lower odds of twice-daily toothbrushing and sugar avoidance. Conclusions: Prenatal intentions were only partly reflected in behaviours at 12 months; after correction for multiple comparisons, intention remained associated only with fluoride toothpaste use and avoidance of night-time bottles.

## 1. Introduction

Early childhood caries (ECC) remains one of the most common chronic diseases of childhood and falls disproportionately on children with limited access to preventive and restorative care [1]. It is defined as the presence of one or more decayed (cavitated or non-cavitated), missing (due to caries) or filled surfaces in any primary tooth of a child under 6 years of age [1]. Its aetiology is multifactorial. Frequent and prolonged exposure of the primary dentition to fermentable carbohydrates plays the central role, alongside oral hygiene, socioeconomic status and access to dental care [2,3]. The consequences reach well beyond the teeth: ECC affects the child’s feeding, development and quality of life, and that of the family as well [1,3]. In Romania, a nationally representative survey found a mean number of decayed, missing and filled teeth (DMFT) of 4.89 in 6-year-old children, with fewer than one in six children caries-free, and noted the absence of national oral health prevention or education programmes [4]; research on parents’ knowledge and practices regarding ECC also remains limited [5].

Among the modifiable risk factors, the best-documented are night-time bottles containing sweetened drinks, frequent intake of added sugar from complementary foods and drinks, late initiation of toothbrushing and insufficient fluoride exposure [2,6,7,8,9]. A systematic review commissioned by the World Health Organization found an association between sugar-containing liquids given by bottle and ECC risk, and showed that breastfeeding at up to 2 years of age does not increase this risk, unlike cariogenic feeding practices [6]. Recent longitudinal studies confirm the link between sweets and sugar-sweetened drinks consumed in the first years of life and subsequent caries [8,9].

Pregnancy and the first year of life are widely regarded as a window of opportunity for the primary prevention of ECC, because this is when the child’s oral hygiene and feeding habits take shape under the direct influence of the parents, especially the mother [10]. Antenatal visits are a natural moment for preventive counselling, given that pregnant women are in fairly regular contact with health professionals [11,12]. Yet studies consistently show that pregnant women have low to moderate knowledge of oral health, both their own and their future child’s, particularly regarding when to start infant oral hygiene and when to schedule the first dental visit [13]. Structural barriers (cost, limited access, lack of active referral to dental services by antenatal staff), together with the belief that dental treatment during pregnancy may be unsafe, further contribute to the underuse of oral health services during this period [11,14]. Community-based and primary care interventions appear to improve maternal knowledge and behaviours and some clinical indicators, but their effect on the child’s oral health remains uncertain because of the variable methodological quality of the available studies [15].

International guidelines recommend starting toothbrushing as soon as the first tooth erupts, using a small, rice-grain-sized amount of fluoride toothpaste from the outset [16,17]. The first dental examination is recommended at the eruption of the first tooth and no later than 12 months of age [18]. These recommendations are not applied consistently, however, even by the professionals who care for women during pregnancy and after birth. In one study of midwives, advice on fluoride was inconsistent and few considered it important to refer the child to a dentist from the first tooth, which may leave parents confused [12].

The literature on prenatal oral health education rarely distinguishes between the stated intention to adopt a behaviour and actually putting it into practice. The Theory of Planned Behaviour (TPB) is the framework most often used to explain this difference: intention is regarded as the most proximal predictor of behaviour and is itself shaped by attitudes, subjective norms and perceived behavioural control [19]. The model has also been applied to children’s oral health, both for toothbrushing and for preventive dental visits [20,21]. Studies based on the TPB and on the Health Action Process Approach (HAPA) have shown that intention, self-efficacy and perceived control are associated with parent-supervised toothbrushing, and that the step from intention to behaviour is mediated by action planning and action control [22]. Interventions targeting these processes, however, have not consistently changed behaviour [23]. Moreover, most studies have been cross-sectional or have followed the intention–behaviour relationship over short periods in children who had already been born. Prospective data comparing intentions formed during pregnancy with what parents actually do by the end of the child’s first year are scarce [24], even though this period is decisive for establishing oral hygiene routines and for the first encounter with the main risk factors for ECC.

In a prospective mother–child cohort, we set out to assess how far the preventive intentions stated by mothers in the third trimester of pregnancy were reflected in the behaviours they reported 12 months after birth. Six behaviours relevant to ECC prevention were examined: starting toothbrushing, brushing frequency, use of fluoride toothpaste, avoidance of added sugar, avoidance of night-time bottles containing anything other than water, and the first dental visit. We also looked at which maternal factors were associated with putting these behaviours into practice. The TPB served as the conceptual framework, but only prenatal intention was measured, not the other constructs of the model. The secondary aim was to explore the relationship between the mother’s oral status during pregnancy, assessed clinically with the International Caries Detection and Assessment System (ICDAS) and the Plaque Control Record (PCR), and the behaviours later adopted for the child. Our hypothesis was that the mother’s own oral hygiene might be associated with these behaviours independently of her knowledge and stated intention. Night-time breastfeeding after the eruption of the first tooth was described separately, without being classified as favourable or unfavourable.

## 2. Materials and Methods

### 2.1. Study Design

We conducted an observational, prospective, longitudinal study of mother–child pairs. The primary aim was to assess how far prenatal intentions regarding the child’s oral health translated into preventive practices during the first year of life, and which maternal factors were associated with these practices. The secondary aim was to explore the relationship between the mother’s oral status at T0 and the preventive practices subsequently adopted.

This study is a secondary observational analysis carried out within a larger project on maternal oral health and the prevention of early childhood caries. Participants were assessed twice: in the third trimester of pregnancy, between gestational weeks 34 and 38 (T0) and when the child was 12 months old or shortly thereafter (T12). The mother’s clinical examination was performed once, at T0. The study is reported in accordance with the Strengthening the Reporting of Observational Studies in Epidemiology (STROBE) guidelines [25].

### 2.2. Sample Size

Sample size was calculated for the paired comparison of intentions at T0 with practices reported at T12, using the McNemar test, following the method described by Connor [26]. Based on published data on the concordance between prenatal intentions and later child oral health behaviours [24], we assumed a conservative scenario with discordant pair proportions of 20% and 8%. With a two-sided significance level of α = 0.05 and 80% power, at least 151 mother–child pairs with complete data were required.

The final analytical sample of 159 mother–child pairs with complete, evaluable data for all six domains (Section 3.1) exceeded this minimum.

### 2.3. Participants and Recruitment

Between April 2023 and May 2024, 218 pregnant women were consecutively recruited from family medicine practices and assessed at the Faculty of Dental Medicine, “George Emil Palade” University of Medicine, Pharmacy, Science, and Technology of Târgu Mureș, Romania. Women aged 18 years or older who agreed to take part, to be examined and to complete the baseline questionnaire, and who signed informed consent, were included. Each participant was assigned a unique code used to link T0 and T12 data.

Participant flow, loss to follow-up and exclusions are reported in Section 3.1.

### 2.4. Baseline Assessment (T0)

The T0 assessment took place in the third trimester of pregnancy and began with a structured online questionnaire on sociodemographic characteristics, knowledge and intentions regarding the future child’s oral health, followed by a plaque assessment and a clinical examination of caries status after professional tooth cleaning.

#### 2.4.1. Plaque Assessment

Dental plaque was assessed first, before any cleaning, with the Plaque Control Record (PCR), following the method described by O’Leary et al. [27]. After application of a disclosing agent, the presence or absence of plaque was recorded on four surfaces of each tooth present: buccal, oral, mesial and distal. PCR was calculated as the percentage of surfaces with plaque out of all surfaces examined: PCR (%) = (number of surfaces with plaque/total number of surfaces examined) × 100. Higher values indicate more plaque. PCR was analysed as a continuous variable; the 50% threshold was used only to describe the sample.

#### 2.4.2. Clinical Assessment of Maternal Caries

After the plaque assessment, professional tooth cleaning was performed and maternal caries status was assessed with the International Caries Detection and Assessment System (ICDAS) [28], following the standard protocol. Teeth were examined visually under standardised conditions, first wet and then after air-drying. All accessible surfaces of each tooth present were inspected and scored according to the standard criteria, from 0 (sound) to 6 (extensive cavity with visible dentine). Each tooth was assigned the highest score recorded on any of its surfaces. Teeth were then grouped into two categories: ICDAS 0–2 (no detectable lesion or initial lesion) and ICDAS 3–6 (moderate or extensive lesion).

#### 2.4.3. T0 Questionnaire

Before the clinical examination, participants completed a structured online questionnaire covering age, educational level and parity (number of previous children). Primiparity was defined as having no other children.

Knowledge of infant oral health was assessed with six statements on the importance of primary teeth, when to start toothbrushing, fluoride toothpaste, sugar exposure, night-time bottles containing potentially cariogenic liquids and the timing of the first dental visit. Response options were “True”, “False” and “Don’t know”. Each correct answer scored one point, giving a total score of 0 to 6, with higher values indicating better knowledge. The score was used as a summative index of correct answers, not as a validated psychometric scale.

The questionnaire was developed by the authors for this study. Knowledge and intention items were based on current international recommendations on infant oral health [16,17,18] and on topics addressed in previous studies of maternal oral health knowledge [13]. The draft was reviewed for content and clarity by all members of the research team, which included paediatric dentists, and revised accordingly. Each intention item at T0 referred to a specific, observable behaviour and was worded in parallel with the corresponding behaviour item at T12. The questionnaire was not formally pretested or psychometrically validated (see Section 4.9). The full questionnaire is provided as Supplementary File S1.

#### 2.4.4. Prenatal Intentions Regarding Preventive Practices

The same questionnaire assessed mothers’ intentions regarding six oral health practices for the child: when to start toothbrushing, brushing frequency, use of fluoride toothpaste, frequency of foods or snacks with added sugar, night-time use of a bottle containing milk, formula, juice or any drink other than water after tooth eruption, and the planned timing of the first dental visit. Night-time breastfeeding was recorded separately from night-time bottle use. Intention items were worded to mirror the T12 items on actual practices, to allow direct comparison.

After the baseline assessment, participants received no educational or preventive intervention as part of this study.

### 2.5. Assessment at 12 Months (T12)

When the child reached 12 months of age or shortly thereafter, participants were contacted by telephone and the follow-up questionnaire was sent to them online. The T12 assessment was carried out between April 2024 and May 2025. At the time of the T12 assessment, the children were aged 12.4 ± 0.7 months (mean ± SD; range 12–15 months). The mother’s clinical examination was not repeated.

The T12 questionnaire covered the following: presence of erupted teeth and the child’s age at eruption of the first tooth, when toothbrushing started, current brushing frequency, use of fluoride toothpaste, frequency of foods or snacks with added sugar during the first year, night-time bottle use after tooth eruption, night-time breastfeeding, whether a first dental visit had taken place and the child’s age at that visit. We also recorded who was mainly responsible for the child’s oral hygiene. Added sugar intake during the first year was reported retrospectively by the mother as usual frequency and therefore does not represent a prospective measure of each exposure. The questionnaire also asked about information on the child’s oral health received after T0 and its source, the perceived usefulness of information received during pregnancy, and any recommendations mothers had been unable to follow; these data were not included in the analyses.

### 2.6. Definition of Favourable Preventive Behaviours

The following behaviours were considered favourable: starting toothbrushing at the eruption of the first tooth; brushing at least twice a day; using fluoride toothpaste; no foods or snacks with added sugar during the first year of life; avoiding night-time bottles containing milk, formula, juice or any drink other than water after the eruption of the first tooth; and a first dental visit by 12 months of age inclusive. This strict definition of added sugar avoidance follows current recommendations to avoid added sugars in children under 2 years of age [29,30] and mirrors the wording of the corresponding T0 intention item.

Night-time breastfeeding after the eruption of the first tooth was assessed separately from night-time bottle use and was not classified as favourable or unfavourable. For added sugar, the answer “I don’t remember” was treated as missing. For the night-time bottle, not using a bottle after the eruption of the first tooth was considered favourable. For the first dental visit, visits made at exactly 12 months were considered favourable. At T0, “Don’t know” answers to intention items were classified as unfavourable intentions, since they do not reflect a formed preventive intention, and the option “I don’t intend to use a bottle” was classified as a favourable intention. At T12, the answers “I don’t know whether the toothpaste contains fluoride” and “I don’t remember” (for the night-time bottle) were treated as missing. Participants with missing or non-evaluable data at T12 for any of the six domains were excluded from the longitudinal analyses.

In this study, a favourable intention is an intention at T0 that corresponds to the recommended practice, and a favourable behaviour is the corresponding recommended practice reported at T12, as defined above. The implementation rate is the proportion of mothers with a favourable intention who reported the corresponding favourable behaviour, and the intention–behaviour gap is the difference between the proportions of favourable intentions and favourable behaviours.

### 2.7. Study Outcomes

The primary outcome was the implementation of preventive oral health behaviours for the child at T12. Each of the six behaviours was analysed separately. For each T0–T12 item pair, participants were assigned to one of four categories: favourable intention and favourable behaviour (F→F), favourable intention and unfavourable behaviour (F→U), unfavourable intention and favourable behaviour (U→F), and unfavourable intention and unfavourable behaviour (U→U).

Longitudinal analyses were performed in the final analytical sample of 159 mother–child pairs with complete, evaluable data for all six domains. For each behaviour, the implementation rate was calculated among participants with a favourable prenatal intention as F→F/(F→F + F→U).

### 2.8. Predictor Variables and Multivariable Models

For the multivariable analysis we selected four maternal variables with a theoretical bearing on the step from intention to behaviour: the prenatal intention corresponding to each behaviour, knowledge score at T0, educational level and primiparity. Education was analysed in two categories: university (including postgraduate) and non-university. The same predictors were used for each of the first five behaviours.

ICDAS, PCR and maternal age were collected as baseline variables and described, but were not included in the main models. In a secondary, exploratory analysis, we fitted an extended model for each of the first five behaviours, in which the presence of at least one tooth with ICDAS 3–6 and PCR were added simultaneously to the four predictors of the main model. PCR was entered as a continuous variable and the association was expressed per 10-percentage-point increase. The extended models were thus adjusted for the corresponding intention, knowledge score, education and primiparity. Maternal age was not included.

### 2.9. Examiner Calibration

Before the start of the study, the two examiners were trained and calibrated in applying the ICDAS criteria and recording the PCR. Inter-examiner agreement was assessed by independent examination of the same 18 participants. For tooth-level ICDAS scores, the weighted kappa was 0.83, and for PCR values the intraclass correlation coefficient (ICC) was 0.89.

### 2.10. Statistical Analysis

Statistical analysis was performed with IBM SPSS Statistics, version 30.0 (IBM Corp., Armonk, NY, USA). Continuous variables were described as mean and standard deviation or median and interquartile range, depending on their distribution, and categorical variables as frequencies and percentages. Baseline characteristics of participants included and not included in the final analysis were compared with the independent-samples t-test for continuous variables and Pearson’s chi-squared test for categorical variables.

Night-time breastfeeding was analysed descriptively only. Proportions were calculated separately at T0 and T12 among participants with evaluable answers at each assessment; “Don’t know” and “Not applicable” answers were excluded from the denominator as appropriate. Night-time breastfeeding was included neither in the McNemar tests nor in the regression models.

Correspondence between intentions at T0 and behaviours at T12 was analysed using the paired F→F, F→U, U→F and U→U matrices, and differences between paired proportions were tested with the exact McNemar test. For each behaviour, we also report the implementation rate among participants with a favourable intention.

For each of the first five behaviours we fitted a separate multivariable logistic regression model including the corresponding prenatal intention, knowledge score, educational level and primiparity. Results are expressed as adjusted odds ratios (aOR) with 95% confidence intervals. The extended models described above additionally included maternal caries status (≥1 tooth with ICDAS 3–6) and PCR; for PCR, the aOR and confidence interval are reported per 10-percentage-point increase. These analyses were considered secondary and exploratory. No multivariable model was fitted for the first dental visit by 12 months, because of the small number of favourable outcomes. Statistical significance was set at *p* < 0.05. The McNemar tests addressed the primary aim; the main regression models were explanatory and the extended models exploratory. No correction for multiple comparisons was planned. In a post hoc Holm correction applied to the *p*-values of the McNemar tests and of the main regression models, all six McNemar comparisons remained significant; in the main regression models, the associations of intention with fluoride toothpaste use and night-time bottle avoidance remained significant, whereas those with starting toothbrushing and twice-daily toothbrushing did not (adjusted *p* = 0.072 and 0.090, respectively). Analyses were performed on complete cases. Multiple imputation was not used because missing values concerned the outcomes themselves, mostly as “I don’t remember” answers, which are unlikely to be missing at random.

### 2.11. Ethical Considerations

The study was conducted in accordance with the Declaration of Helsinki and was approved by the Ethics Committee of the “George Emil Palade” University of Medicine, Pharmacy, Science, and Technology of Târgu Mureș (No. 2169/09.03.2023, approved on 9 March 2023). All participants received written and verbal information about the study and signed informed consent before inclusion. Data were coded and handled confidentially.

## 3. Results

### 3.1. Characteristics of the Study Population

Of the 218 pregnant women initially enrolled in the third trimester, 177 completed follow-up at 12 months postpartum (retention 81.2%) and 41 (18.8%) were lost to follow-up because they did not complete the T12 questionnaire. A further 18 participants were excluded from the longitudinal analyses because they had skipped at least one question or answered “I don’t remember” in at least one of the six behavioural domains. All children had at least one erupted tooth at T12. The final analytical sample therefore comprised 159 mother–child pairs with complete, evaluable data for all six domains (Figure 1). The 159 participants included in the final analysis did not differ significantly from the 59 who were not included in age, parity, knowledge score, caries status, PCR or any of the six prenatal intentions. Non-included participants were less-often university-educated (47.5% vs. 60.4%), although this difference was not statistically significant (*p* = 0.087; Table S1).

Mothers in the final sample had a mean age of 29.4 ± 4.8 years, and 92 (57.9%) were primiparous. The mean knowledge score was 4.0 ± 1.3 out of 6. At the baseline examination, 117 participants (73.6%) had at least one tooth with an ICDAS score of 3–6. Mean PCR was 48.0 ± 19.0%, and 75 participants (47.2%) had a PCR of 50% or higher. Ninety-six participants (60.4%) had a university education and 63 (39.6%) did not (Table 1).

### 3.2. Prenatal Preventive Intentions

During pregnancy, the most frequent favourable intentions concerned avoiding added sugar in the first year (86.8%), brushing at least twice a day (83.0%) and starting toothbrushing when the first tooth erupted (82.4%). Overall, 67.9% of participants intended to use fluoride toothpaste and 65.4% intended to avoid night-time bottles containing anything other than water after the first tooth erupted. Only 38.4% planned a first dental visit by 12 months of age (Table 2).

### 3.3. Correspondence Between Intentions and Behaviours at T12

The largest differences between favourable proportions at T0 and T12 were seen for avoiding added sugar (86.8% vs. 40.3%), brushing at least twice a day (83.0% vs. 38.4%) and starting toothbrushing at the eruption of the first tooth (82.4% vs. 51.6%). A first dental visit by 12 months had been planned by 38.4% of participants but was reported at T12 by only 10.1%. For the night-time bottle, the proportions were 65.4% at T0 and 56.0% at T12; for fluoride toothpaste, they were 67.9% and 58.5% (Table 3).

#### Individual Concordance Between Intentions and Behaviours

Implementation rates among participants with a favourable intention ranged from 21.3% for the first dental visit to 81.5% for fluoride toothpaste and were below 50% for twice-daily brushing and for avoidance of added sugar (Table 4).

Differences between intentions and behaviours were significant in all six domains (exact McNemar test, *p* ≤ 0.032; Table 4), and in every domain transitions from a favourable intention to an unfavourable behaviour were more frequent than transitions in the opposite direction.

### 3.4. Maternal Factors Associated with Implementation of Preventive Practices

Separate multivariable logistic regression models were fitted for the first five behaviours, each including the corresponding prenatal intention, knowledge score, educational level and primiparity. No multivariable model was fitted for the first dental visit, because only 16 of the 159 participants reported this behaviour.

After adjustment, a favourable prenatal intention was independently associated with starting toothbrushing at the eruption of the first tooth, brushing at least twice a day, using fluoride toothpaste and avoiding night-time bottles, but not with avoidance of added sugar (Table 5). After correction for multiple comparisons, only the associations with fluoride toothpaste use and avoidance of night-time bottles remained significant. Knowledge score, educational level and primiparity were not significantly associated with any of the five behaviours modelled.

In the extended models, having at least one tooth with ICDAS 3–6 at T0 was not significantly associated with any behaviour at T12. In these exploratory models, a higher maternal PCR at T0 was associated with a lower likelihood that the child’s teeth were brushed at least twice a day at 12 months and a lower likelihood that added sugar was avoided during the first year (Table 6).

### 3.5. Night-Time Breastfeeding

Night-time breastfeeding after the eruption of the first tooth was analysed descriptively and separately from night-time bottle use. During pregnancy, 98 of the 150 participants with evaluable answers (65.3%) intended to continue night-time breastfeeding after the first tooth erupted; seven answered “Don’t know” and two chose “Not applicable”. At T12, 46 of the 110 participants for whom the question applied (41.8%) were breastfeeding at night, 27 (24.5%) occasionally and 19 (17.3%) several times a night; the remaining 64 were not, and another 49 chose “Not applicable”.

## 4. Discussion

### 4.1. Summary of Main Findings

We compared the preventive intentions stated during pregnancy with the behaviours reported at 12 months postpartum for six aspects of children’s oral health. Most mothers reported favourable intentions for five of them (from 65.4% for avoiding night-time bottles to 86.8% for avoiding added sugar), but only 38.4% planned a dental visit by 12 months. At 12 months, the proportion of favourable behaviours was significantly lower than that of favourable intentions in all six domains (*p* < 0.05; Table 4) and shifts from “favourable” to “unfavourable” were more common than the reverse. The implementation rate, that is, the percentage of mothers with a favourable intention who went on to adopt the behaviour, ranged from 21.3% for the first dental visit to 81.5% for fluoride toothpaste (Table 4). The differences between T0 and T12 were about nine percentage points for fluoride toothpaste (67.9% vs. 58.5%) and the night-time bottle (65.4% vs. 56.0%) but exceeded 44 points for twice-daily brushing (83.0% vs. 38.4%) and avoidance of sugar (86.8% vs. 40.3%) (Table 3). The intention–behaviour gap, well described in the health-behaviour literature [31], is therefore not uniform; it depends on how complex each behaviour is and on the barriers it involves. The correspondence between behaviours planned during pregnancy and those carried out has also been examined in a prospective study of first-time mothers [24].

### 4.2. Preventive Behaviours: Starting Toothbrushing, Brushing Frequency and Avoiding Night-Time Bottles

For starting toothbrushing at the eruption of the first tooth (58.0% implementation) and avoiding night-time bottles (72.1%), prenatal intention remained significantly associated with the behaviour after adjustment for knowledge score, educational level and primiparity (Table 5), although for starting toothbrushing this association did not remain significant after correction for multiple comparisons. This is in line with studies based on the TPB and HAPA, which have shown the importance of parental intention for the oral hygiene of young children [21,22]. Unlike those studies, however, we measured only intention, not attitudes, subjective norms or perceived control.

One possible explanation is that both behaviours fit easily into a family routine. They do not require repeated decisions throughout the day; they can be set up in advance as rules tied to moments of the day. Research on action planning suggests that such strategies can help turn intentions into behaviour [22], although in a recent intervention study better planning did not increase the frequency of supervised brushing [23]. As we did not assess these mechanisms directly, this explanation remains a hypothesis.

Brushing at least twice a day was achieved by 42.4% of the mothers who had planned it, even though intention was significantly associated with the behaviour in the main model (Table 5); this association, however, did not remain significant after correction for multiple comparisons. Unlike simply starting to brush, keeping up an adequate frequency means repeating the behaviour every day. Our results suggest that starting a routine and sustaining it are different challenges for parents, but the study did not assess what influenced adherence to the recommended frequency.

#### Night-Time Breastfeeding Versus Night-Time Bottle Use

Night-time breastfeeding was kept separate from night-time bottle use because the evidence on the two practices differs: breastfeeding up to 2 years of age does not increase ECC risk, unlike night-time bottles containing milk, formula, juice or any liquid other than water [6], so grouping them could have led to misleading interpretations. About two-thirds of mothers intended to continue night-time breastfeeding after the first tooth erupted, and at T12 fewer than half of those for whom the question applied were doing so (Section 3.5). These proportions cannot be compared directly, because they have different denominators and individual concordance was not analysed. As we did not examine the children’s teeth, these data do not allow conclusions about the link between night-time breastfeeding and ECC.

### 4.3. Added Sugar: The Intention–Behaviour Gap and the Absence of a Significant Adjusted Association

Avoiding added sugar was the most common favourable intention at T0 (86.8%) but had one of the lowest implementation rates (43.5%), and in the adjusted model intention was not significantly associated with behaviour (Table 5). The low rate should also be read in light of the strict definition we used, which required no foods or snacks with added sugar at all during the first year, and of the retrospective nature of the reporting. Because this binary definition places mothers whose children received added sugar occasionally and those whose children received it daily in the same unfavourable category, the low rate reflects a failure to meet a strict recommendation rather than evidence of frequent sugar exposure. Longitudinal studies consistently show that sweets and sugar-sweetened drinks introduced with complementary feeding increase ECC risk [8,9]. Limiting sugar, however, is not a one-off decision; it involves daily choices shaped by social pressure, convenience and whatever products are at hand. We did not measure these factors, so this explanation remains speculative. The WHO-commissioned systematic review also judged the evidence on many dietary risk factors for ECC to be of limited quality [6], which underlines the need for longitudinal studies that capture real dietary exposure more accurately. One possible explanation, which we could not test, is that an intention formed during pregnancy may not anticipate the pressures that arise later, with complementary feeding, family dynamics or the influence of other relatives. In this respect, sugar differs both from behaviours built into the daily routine (Section 4.2) and from the single act of a first dental visit (Section 4.5).

### 4.4. Fluoride Toothpaste

Fluoride toothpaste had the highest implementation rate of the six behaviours (81.5%; Table 4) and the strongest association with prenatal intention (aOR 36.20; 95% CI: 12.40–105.70; Table 5). Only five of the 51 participants without a favourable intention (9.8%) were using fluoride toothpaste at 12 months. The very wide confidence interval, however, shows that the estimate is imprecise because of the small numbers in some categories, so the exact size of the effect should be interpreted with caution. The estimate nevertheless reflects a very strong concordance in the observed data: the crude odds ratio calculated from the counts in Table 4 was 40.5, close to the adjusted value, so the direction and consistency of the association are more informative than its exact magnitude.

A possible explanation, not tested in this study, lies in the nature of the behaviour. Twice-daily brushing and limiting sugar demand daily effort, whereas using fluoride toothpaste depends mainly on a single decision, choosing the product, which is then repeated automatically at every brushing. Recommendations on fluoride toothpaste are also fairly uniform and widely known (a rice-grain-sized amount from the first tooth) [16,17], and the cost is low. A single, inexpensive decision that slots easily into an existing routine may translate more readily from intention into practice.

The group without a favourable intention also included mothers who answered “Don’t know” at T0. For this behaviour, intention may therefore also reflect how well-informed the mother was about fluoride, not only her motivation. The fact that very few of these mothers later used fluoride toothpaste may indicate that information received after birth did not make up for this initial gap, although we did not analyse this. Inconsistent advice from health professionals on fluoride use in young children may also play a part [12]. Explicit antenatal counselling on fluoride toothpaste could therefore be a simple intervention with considerable potential.

### 4.5. First Dental Visit: The Lowest Implementation Rate

The first dental visit had the lowest proportion of favourable intentions at T0 (38.4%) and the lowest frequency at T12 (10.1%; 16/159; Table 3). Of the 61 mothers who had planned a visit by 12 months, only 13 attended one (21.3%; Table 4). Unlike the other behaviours, this is not a daily routine but a single act that depends on access to services: getting an appointment, cost, the availability of a paediatric dental practice and time. Studies on the use of dental services around pregnancy show that, besides the mother’s attitudes and beliefs [14], cost, distance, lack of referral by antenatal staff and educational level all matter [11,32,33]. In these studies, women with higher education and those who took part in health education activities during pregnancy were more likely to attend a dental check-up [11,33]. In a TPB-based study, parental intention likewise did not predict actual attendance of preschool children at preventive check-ups [20]. In addition, few midwives regard referral to a dentist from the first tooth as very important, far fewer than those who recommend fluoride toothpaste [12]. This could partly explain why an intention stated during pregnancy so rarely turned into a visit; however, we did not analyse the information and guidance that mothers received from the health system. Several other barriers, none of which were measured in this study, may also have contributed: parents’ lack of awareness that a first visit is recommended by 12 months of age (the low proportion of mothers who planned such a visit, 38.4%, itself suggests a knowledge gap), cost, the limited availability of dental services for infants, the concentration of dental practices in urban areas, and the absence of routine referral by family physicians, paediatricians and midwives. The small number of events (16/159) did not allow us to fit a multivariable model, so we could not identify the factors associated with a first dental visit. Larger future studies focused on barriers to access could answer this question.

### 4.6. The Role of Maternal Knowledge, Education and Parity

None of the adjusted models showed a significant association between knowledge score, educational level or primiparity and any of the behaviours (Table 5). This runs counter to the premise behind many prenatal education programmes, which rely mainly on providing information [32], namely that more knowledge leads directly to better behaviour. It is, however, consistent with the TPB, in which knowledge influences behaviour mainly indirectly, through attitudes, perceived control and intention [19]. In our sample, knowledge was not associated with behaviour. This is consistent with the view that information alone may not be enough and that interventions may also need to support planning and action [22,34]. Education was not significantly associated with any of the behaviours either, although other studies have linked lower maternal education with lower use of dental services or less-frequent toothbrushing in the child [33,35]. It should be noted, though, that the knowledge score was a simple six-item index without psychometric validation, and that education was analysed in only two categories (university or not), which may have masked real differences.

The absence of associations for these three factors may also reflect the limited statistical power of the sample (*n* = 159) rather than a true absence of effect, a point we return to in the limitations.

### 4.7. Maternal Oral Status and the Child’s Behaviours: An Unexpected Association

The extended exploratory analyses (Table 6) yielded an unexpected finding. A higher maternal PCR at T0 was associated with a lower likelihood that the child was brushed at least twice a day at 12 months and a lower likelihood that added sugar was avoided. In this sample, the mother’s objectively measured oral hygiene was thus associated with the behaviours adopted for the child, independently of knowledge and stated intention. Because of the observational design, this association does not show that the mother’s oral hygiene directly influences the behaviours adopted for the child. This fits with studies showing that parents’ oral health habits are reflected in their children’s oral health [10] and with recent longitudinal data in which parents’ own toothbrushing at 12 months predicted the child’s brushing frequency at 2 years [35]. One hypothesis is that habits are passed on through everyday example rather than through information; our design does not allow this to be tested, and the association may also reflect unmeasured factors, such as socioeconomic conditions or general health awareness. A mother with poor oral hygiene may, however well-informed she is, have a less consistent care routine for herself and for her child, although the study design does not allow this hypothesis to be tested. Having a tooth with ICDAS 3–6 was not significantly associated with any of the child’s behaviours. One explanation may be that PCR reflects current hygiene, and therefore behaviour, whereas ICDAS reflects accumulated caries history. Because several models were tested, these associations should be regarded as hypothesis-generating and need to be confirmed in future studies.

### 4.8. Clinical Implications

Our data show that the gap between prenatal intention and practice was largest for behaviours that must be repeated daily (twice-daily brushing, avoidance of added sugar) and for the first dental visit, that prenatal intention was associated with four of the five behaviours modelled (two of which remained significant after correction for multiple comparisons), and that knowledge, education and parity were not. They also suggest, in exploratory analyses, an association between poorer maternal oral hygiene and less favourable behaviours for the child. Our study did not evaluate any intervention. The following suggestions are therefore based on existing evidence and guidelines and are consistent with, but not demonstrated by, our findings: interventions that support action planning may help with daily behaviours [22]; assisted appointment booking or explicit referral by antenatal and primary care professionals, among the few who see pregnant women regularly, may increase early dental visits [11,12,15]; and clear, consistent counselling on fluoride toothpaste during pregnancy, in line with current guidelines [16,17], may be a simple and inexpensive measure. Whether including the mother’s own oral health in perinatal programmes improves children’s behaviours remains to be tested.

### 4.9. Limitations

This study has several limitations. Behaviours at 12 months were reported retrospectively by the mothers, without an objective measure. Self-reporting may be influenced by social desirability, although the size of this effect varies widely with the context and data collection method [36,37]. Brushing frequency, the use of fluoride toothpaste and the child’s diet are particularly prone to social desirability and recall bias, because they are repeated daily, were recalled over a whole year, and mothers are generally aware of what is recommended; in addition, mothers may not always have known whether the toothpaste they used contained fluoride. Both biases would most likely lead to over-reporting of favourable behaviours; if so, the true gap between prenatal intentions and actual practice would be larger than observed, and our estimates should be regarded as conservative. These biases may also have affected the associations with prenatal intention: mothers who had stated a favourable intention may have been more inclined to report the corresponding behaviour at 12 months, for consistency with their earlier answers, which could have inflated the observed associations. Simply asking mothers about their intentions at T0 may have influenced their later behaviour (the question–behaviour effect), although this effect is usually small [38]. Although we used the TPB as a framework, we measured only intention, not attitudes, subjective norms or perceived control, so we could not examine the mechanisms by which intention did or did not translate into behaviour. The knowledge score is not a validated scale, and the questionnaire was not formally pretested. The intention and behaviour items were not psychometrically evaluated, but they were factual, based on international recommendations and mirrored across the two assessments. Sugar intake was reported retrospectively as usual frequency during the first year and assessed against a strict criterion (no intake at all), which may introduce recall error and obscure partial adherence to recommendations. The timing of the first exposure to added sugar was not recorded. Oral health advice received after birth, for example from paediatricians or family physicians, was recorded but not analysed. Such advice may have helped some mothers to adopt favourable behaviours regardless of their prenatal intention, and its absence may have contributed to the gap observed in others. “Don’t know” answers on intentions were treated as unfavourable, which may underestimate the proportion of favourable intentions. Analyses were restricted to participants with complete data. Participants who were not included (41 lost to follow-up and 18 excluded) did not differ significantly from the final sample in any baseline characteristic, including prenatal intentions, although they were somewhat less-often university-educated (Table S1); this reduces, but does not exclude, the possibility of attrition bias. If mothers lost to follow-up or unable to recall their practices had less favourable behaviours, favourable behaviours may have been overestimated and the intention–behaviour gap underestimated. The retention rate (81.2%) is typical for longitudinal studies of this kind, and the analytical sample exceeded the minimum required size.

We performed multiple tests (six McNemar tests and ten logistic regression models), reflecting the number of behaviours examined. No correction for multiple comparisons was planned; the results of a post hoc Holm correction are reported in Section 2.10. The results should be interpreted with this in mind, particularly the associations from the extended analyses (Table 6), which are exploratory and hypothesis-generating and need to be confirmed in larger samples. For the first dental visit, the small number of events (16/159) did not allow for a multivariable model. The analysis of night-time breastfeeding was descriptive only, and the proportions at the two assessments were calculated in different populations. We did not assess system-level factors specific to the local setting, such as access to paediatric dentistry or costs, which might explain the low rate of first dental visits. In Romania, dental care is delivered mainly in the private sector and dental practices are concentrated in urban areas [4]. The high proportion of participants with a university education (60.4%) suggests possible selection bias, as the sample was probably better informed and more motivated than the general population of pregnant women. Finally, the single-centre observational design suits the exploratory aims of the study but limits causal inference and the generalisability of the findings to other populations or antenatal care systems.

## 5. Conclusions

In this prospective cohort, favourable behaviours at 12 months postpartum were significantly less frequent than the corresponding prenatal intentions in all six preventive domains, with the largest gaps for twice-daily toothbrushing and avoidance of added sugar; only 10.1% of children had seen a dentist by 12 months. After correction for multiple comparisons, prenatal intention remained independently associated only with fluoride toothpaste use and avoidance of night-time bottles, and it was not associated with avoidance of added sugar. Maternal knowledge, educational level and parity were not significantly associated with any of the behaviours modelled.

## Figures and Tables

**Figure 1:**
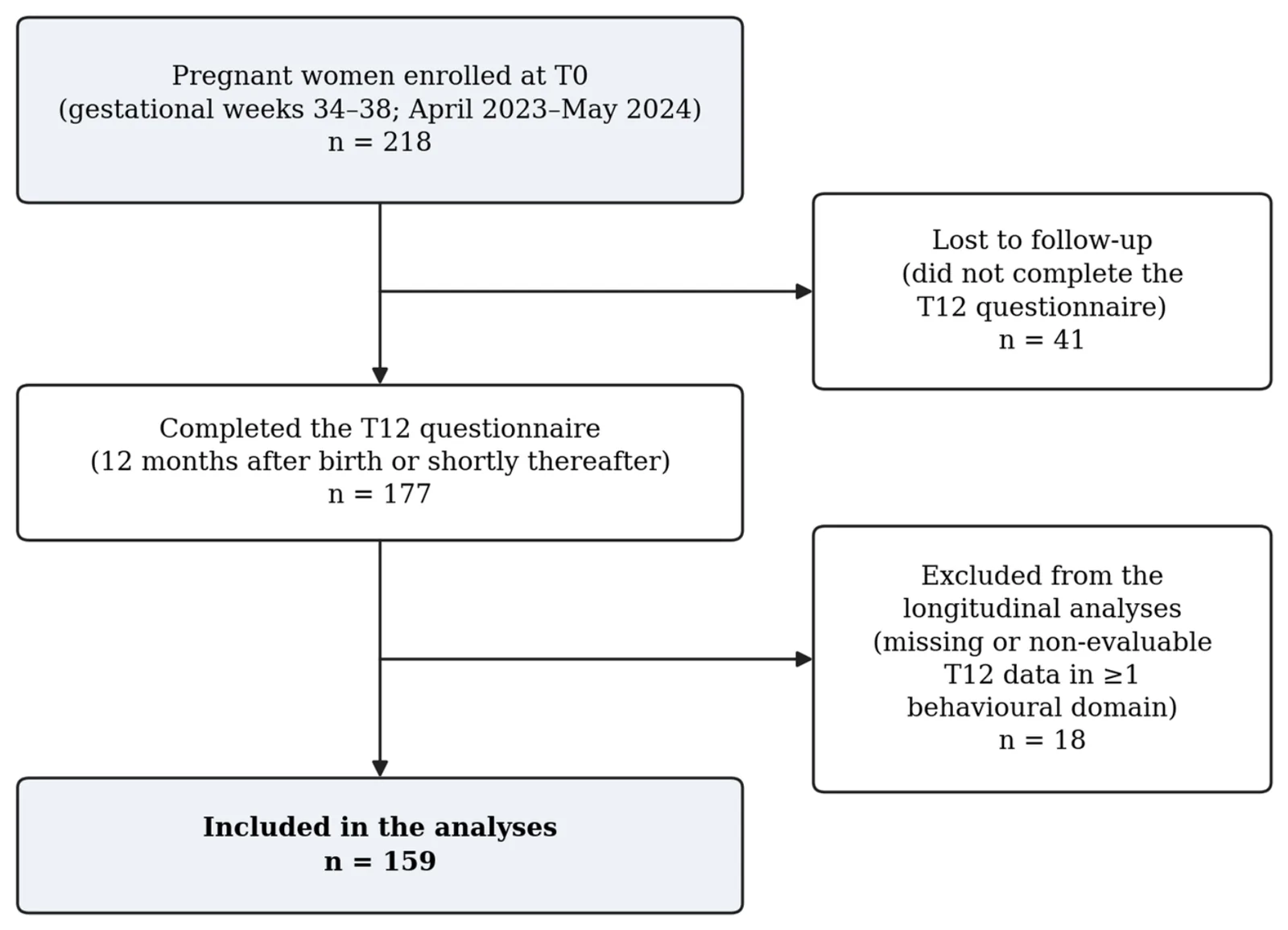
Flow of participants through the study.

**Table 1:** Baseline characteristics of participants included in the final analytical sample.

Characteristic	Participants in the Final Sample (*n* = 159)
Maternal age, years, mean ± SD	29.4 ± 4.8
Primiparous, *n* (%)	92 (57.9)
Knowledge score (0–6), mean ± SD	4.0 ± 1.3
≥1 tooth with ICDAS 3–6, *n* (%)	117 (73.6)
No teeth with ICDAS 3–6, *n* (%)	42 (26.4)
PCR, %, mean ± SD	48.0 ± 19.0
PCR ≥ 50%, *n* (%)	75 (47.2)
University education, *n* (%)	96 (60.4)
Non-university education, *n* (%)	63 (39.6)

SD = standard deviation; PCR = Plaque Control Record. The PCR ≥ 50% threshold was used for descriptive purposes only; PCR values were otherwise kept as a continuous variable.

**Table 2:** Favourable preventive intentions at T0.

Behaviour	*n*/159	%
Toothbrushing from the first tooth	131	82.4
Toothbrushing ≥ 2 times/day	132	83.0
Fluoride toothpaste	108	67.9
No added sugar in the first year	138	86.8
No night-time bottle	104	65.4
First dental visit ≤ 12 months	61	38.4

**Table 3:** Intentions at T0 and behaviours reported at T12.

Behaviour	Intention at T0, *n* (%)	Behaviour at T12, *n* (%)
Toothbrushing from the first tooth	131 (82.4)	82 (51.6)
Toothbrushing ≥ 2 times/day	132 (83.0)	61 (38.4)
Fluoride toothpaste	108 (67.9)	93 (58.5)
No added sugar in the first year	138 (86.8)	64 (40.3)
No night-time bottle	104 (65.4)	89 (56.0)
First dental visit ≤ 12 months	61 (38.4)	16 (10.1)

**Table 4:** Paired intention–behaviour matrices, implementation rates and exact McNemar test results.

Behaviour	F→F	F→U	U→F	U→U	Implementation Among F (%)	*p* (Exact McNemar)
Toothbrushing from the first tooth	76	55	6	22	58.0	<0.001
Toothbrushing ≥ 2 times/day	56	76	5	22	42.4	<0.001
Fluoride toothpaste	88	20	5	46	81.5	0.004
No added sugar	60	78	4	17	43.5	<0.001
No night-time bottle	75	29	14	41	72.1	0.032
First visit ≤ 12 months	13	48	3	95	21.3	<0.001

**Table 5:** Maternal factors associated with preventive practices at T12.

**Favourable Behaviour at T12**	**Predictor**	**aOR (95% CI)**	***p***
Toothbrushing from the first tooth (82/159)	Favourable intention at T0	3.18 (1.16–8.69)	0.024
	Knowledge score at T0, per point	1.15 (0.88–1.50)	0.305
	University education	1.31 (0.65–2.63)	0.447
	Primiparity	0.92 (0.46–1.83)	0.811
Toothbrushing ≥2 times/day (61/159)	Favourable intention at T0	2.51 (1.02–6.17)	0.045
	Knowledge score at T0, per point	1.18 (0.90–1.54)	0.230
	University education	1.36 (0.68–2.74)	0.385
	Primiparity	0.86 (0.43–1.71)	0.669
Fluoride toothpaste (93/159)	Favourable intention at T0	36.20 (12.40–105.70)	<0.001
	Knowledge score at T0, per point	1.30 (0.98–1.75)	0.071
	University education	1.20 (0.56–2.58)	0.640
	Primiparity	0.90 (0.42–1.91)	0.780
No added sugar (64/159)	Favourable intention at T0	2.37 (0.87–6.48)	0.098
	Knowledge score at T0, per point	1.17 (0.90–1.53)	0.244
	University education	1.30 (0.65–2.61)	0.458
	Primiparity	0.90 (0.45–1.79)	0.760
No night-time bottle (89/159)	Favourable intention at T0	6.29 (3.08–12.82)	<0.001
	Knowledge score at T0, per point	1.12 (0.86–1.46)	0.402
	University education	1.21 (0.60–2.43)	0.590
	Primiparity	0.84 (0.42–1.67)	0.617

**Table 6:** Association between maternal oral status at T0 and the child’s preventive practices at T12.

Favourable Behaviour at T12	Maternal Clinical Predictor at T0	aOR (95% CI)	*p*
Toothbrushing from the first tooth (82/159)	≥1 tooth with ICDAS 3–6	0.81 (0.38–1.72)	0.580
	PCR, per 10% increase	0.91 (0.80–1.04)	0.170
Toothbrushing ≥2 times/day (61/159)	≥1 tooth with ICDAS 3–6	0.73 (0.34–1.57)	0.420
	PCR, per 10% increase	0.86 (0.75–0.99)	0.041
Fluoride toothpaste (93/159)	≥1 tooth with ICDAS 3–6	0.80 (0.35–1.82)	0.600
	PCR, per 10% increase	0.92 (0.79–1.07)	0.280
No added sugar (64/159)	≥1 tooth with ICDAS 3–6	0.69 (0.32–1.49)	0.350
	PCR, per 10% increase	0.84 (0.73–0.97)	0.019
No night-time bottle (89/159)	≥1 tooth with ICDAS 3–6	0.88 (0.41–1.88)	0.740
	PCR, per 10% increase	0.90 (0.79–1.03)	0.130

Note: Each model was additionally adjusted for the corresponding prenatal intention, knowledge score at T0, educational level and primiparity. PCR was entered as a continuous variable; the aOR corresponds to a 10-percentage-point increase.

## Data Availability

The data presented in this study are available on request from the corresponding author due to privacy restrictions.

## Supplementary Materials

The following supporting information can be downloaded at: https://www.mdpi.com/article/10.3390/healthcare14213652/s1. File S1: Questionnaires T0–T12 (English translation of the original Romanian instrument). File S2: STROBE checklist. Table S1: Baseline characteristics of participants included and not included in the final analysis.

## Abbreviations

The following abbreviations are used in this manuscript:

aOR	adjusted odds ratio
CI	confidence interval
DMFT	decayed, missing and filled teeth
ECC	early childhood caries
HAPA	Health Action Process Approach
ICC	intraclass correlation coefficient
ICDAS	International Caries Detection and Assessment System
PCR	Plaque Control Record
SD	standard deviation
STROBE	Strengthening the Reporting of Observational Studies in Epidemiology
T0	baseline assessment in the third trimester of pregnancy (gestational weeks 34–38)
T12	follow-up assessment at 12 months after birth or shortly thereafter
TPB	Theory of Planned Behaviour
WHO	World Health Organization
