# Supplementary material for: From Prenatal Intention to Postpartum Practice: A Prospective Cohort Study of Oral Health Preventive Behaviours in Mother–Child Pairs
Source: Healthcare (Basel). 2026 Oct 28;14(21):3652. doi: 10.3390/healthcare14213652 (PMC13762710; doi:10.3390/healthcare14213652)
Supplement: Supplementary file 1 [file healthcare-14-03652-s001.zip › File_S1_Questionnaires_T0_T12.pdf]

QUESTIONNAIRES T0–T12
Prospective mother–child study (English translation of the original Romanian instrument)

T0 QUESTIONNAIRE – DURING PREGNANCY

Participant code: ______________________ Date: ____ / ____ / ______ Gestational week: ______

A. General information

1. Age: ______ years

2. Is this the first pregnancy to reach this stage? ☐ Yes ☐ No

3. Do you have other children? ☐ No ☐ Yes, number: ______

4. Education: ☐ lower secondary ☐ upper secondary ☐ post-secondary ☐ university ☐ postgraduate

B. Intentions regarding the child's oral care

1. When do you intend to start brushing your child's teeth?

☐ as soon as the first tooth erupts

☐ after several teeth have erupted

☐ after 1 year of age

☐ don't know

2. Once the first tooth has erupted, how often do you think you will brush your child's teeth?

☐ less than once a day

☐ once a day

☐ twice a day or more

☐ don't know

3. Do you think you will use fluoride toothpaste for your child after the first tooth erupts?

☐ Yes

☐ No

☐ Don't know

4. During the first year of life, how often do you think you will give your child foods or snacks with added sugar?

☐ never

☐ less than once a week

☐ 1–3 times a week

☐ daily

☐ don't know

5. After the first tooth erupts, do you think you will give your child a bottle at night containing milk, formula, juice or any drink other than water?

☐ never

☐ sometimes

☐ often

☐ don't know

☐ I don't intend to use a bottle

6. At what age do you intend to take your child to the dentist for the first time?

☐ By 12 months of age, inclusive

☐ After 12 months of age

☐ only if a problem arises

☐ don't know

Additional question on night-time breastfeeding (analysed separately from bottle use):

7. If you breastfeed after the first tooth erupts, do you think breastfeeding will continue at night?

☐ No

☐ Yes, occasionally

☐ Yes, several times a night

☐ Don't know

☐ Not applicable

C. Basic knowledge of children's oral health

For each statement, tick one option: True / False / Don't know.

1. Primary teeth do not need the same attention to prevent caries, because they will be replaced.

☐ True ☐ False ☐ Don't know

2. Brushing the child's teeth can be postponed until one year of age.

☐ True ☐ False ☐ Don't know

3. Fluoride toothpaste can be used in young children in an age-appropriate amount.

☐ True ☐ False ☐ Don't know

4. Foods with added sugar are recommended in the child's diet before one year of age.

☐ True ☐ False ☐ Don't know

5. Night-time use of a bottle containing milk, formula, juice or drinks other than water, after the teeth have erupted, can increase the risk of caries.

☐ True ☐ False ☐ Don't know

6. The first dental visit should take place by 1 year of age, inclusive.

☐ True ☐ False ☐ Don't know

D. Maternal clinical examination at T0

This section is completed by the examiner, not by the participant. ICDAS is recorded at surface level and the Plaque Control Record (PCR) full-mouth.

ICDAS: separate clinical chart.

PCR (%): ___________

For PCR, each examined surface is scored as 0 = no visible plaque or 1 = visible plaque. PCR (%) = (number of surfaces with visible plaque / total number of surfaces examined) × 100.

T12 QUESTIONNAIRE – THE CHILD AT 12 MONTHS

Participant code: ______________________ Date: ____ / ____ / ______ Child's age: ______ months

A. Information about the child

1. Does your child have any erupted teeth? ☐ No ☐ Yes

2. At what age did the first tooth erupt? ______ months

3. Who is mainly responsible for your child's oral hygiene? ☐ mother ☐ father ☐ both ☐ other person: ___________

B. Practices actually adopted

1. When did you start brushing your child's teeth?

☐ as soon as the first tooth erupts

☐ after several teeth have erupted

☐ I haven't started yet

☐ my child has no teeth yet

2. How often do you currently brush your child's teeth?

☐ less than once a day

☐ once a day

☐ twice a day or more

☐ not applicable – my child has no teeth yet

3. Do you use fluoride toothpaste for your child?

☐ Yes

☐ No

☐ I don't know whether the toothpaste contains fluoride

☐ not applicable – my child has no teeth yet

4. During the first year of life, how often did you give your child foods or snacks with added sugar?

Instruction for the participant: If the frequency varied during the first year, choose the option that best describes the usual frequency**.**

☐ never

☐ less than once a week

☐ 1–3 times a week

☐ daily

☐ I don't remember

5. After the first tooth erupted, did you ever give your child a bottle at night containing milk, formula, juice or any drink other than water?

☐ never

☐ sometimes

☐ often

☐ Not applicable – my child did not use a bottle after the first tooth erupted.

☐ not applicable – my child has no teeth yet

☐ I don't remember

6. Has your child been seen by a dentist before this assessment?

☐ Yes

☐ No

If yes, at what age? ______ months

CODING NOTE (research team): a visit made at 12 months of age is classified as favourable (≤12 months). For children assessed before 12 months of age without a visit, the outcome "visit by 12 months" cannot yet be determined and is not automatically coded as unfavourable.

Reason for the first visit: ☐ preventive check-up ☐ recommended by a doctor ☐ dental/oral problem ☐ other reason: ___________

Additional question on night-time breastfeeding (analysed separately from bottle use):

7. After the first tooth erupted, is your child breastfed at night?

☐ No

☐ Yes, occasionally

☐ Yes, several times a night

☐ Not applicable

C. Exposure to information after T0

8. Since joining the study, have you received any further information on your child's oral health? ☐ No ☐ Yes

If yes, from: ☐ dentist ☐ paediatrician ☐ family doctor ☐ obstetrician ☐ internet/social media ☐ family/friends ☐ other sources: ___________

9. How useful did you find the information you received during pregnancy? 0 1 2 3 4 5 6 7 8 9 10

0 = not at all useful; 10 = extremely useful

10. Was there any recommendation you wanted to follow but were unable to? ☐ No ☐ Yes

If yes, which one? ____

____

NOTE FOR CODING AND ANALYSIS

This section is intended for the research team and need not be included in the form given to participants.

- **Starting toothbrushing:** favourable = brushing started as soon as the first tooth erupted
- **Brushing frequency:** favourable = twice a day or more
- **Fluoride toothpaste:** favourable = use of fluoride toothpaste; the answer “I don't know whether the toothpaste contains fluoride” (T12) is coded as missing
- **Added sugar: favourable = “never” during the first year of life; other frequencies are retained for secondary analyses. The answer “I don't remember” is coded as missing, not as unfavourable behaviour. If the frequency varied, the answer reflects the usual frequency recalled retrospectively, not exact cumulative intake.**
- **Cariogenic night-time bottle:** favourable = never after the first tooth erupted; water is not considered a cariogenic exposure; “I don't intend to use a bottle” (T0) and “Not applicable – my child did not use a bottle after the first tooth erupted” (T12) are coded as favourable; “I don't remember” (T12) is coded as missing
- **First dental visit: favourable = visit at ≤12 months of age; no visit and assessment before 12 months = outcome not yet determinable.**

Night-time breastfeeding is kept as a separate variable and is not automatically combined with bottle use into a single binary outcome. This separation reduces the risk of misclassifying night-time exposures.

PCR should be analysed mainly as a continuous variable (0–100%), not through an arbitrary “good/bad” threshold. ICDAS codes can be recorded in full at data collection and grouped later; in the analysis, teeth were grouped as ICDAS 0–2 and ICDAS 3–6.

T0 intentions: for the six main behaviours, the answer “Don't know” is coded as an unfavourable intention, as it does not reflect a formed preventive intention. The implementation rate is calculated for each behaviour as F→F / (F→F + F→U) among participants with a favourable intention at T0. Participants with missing or non-evaluable T12 data for any of the six domains (answers “I don't remember” or “I don't know whether the toothpaste contains fluoride”, “not applicable” because of absent erupted teeth, or an undeterminable dental visit) are excluded from the longitudinal analyses.
