# Supplementary material for: From Prenatal Intention to Postpartum Practice: A Prospective Cohort Study of Oral Health Preventive Behaviours in Mother–Child Pairs
Source: Healthcare (Basel). 2026 Oct 28;14(21):3652. doi: 10.3390/healthcare14213652 (PMC13762710; doi:10.3390/healthcare14213652)
Supplement: Supplementary file 1 [file healthcare-14-03652-s001.zip › File_S2_STROBE_checklist.pdf]

STROBE Statement — Checklist of items that should be included in reports of cohort studies

Manuscript: "From Prenatal Intention to Postpartum Practice: A Prospective Cohort Study of Oral Health Preventive Behaviours in Mother–Child Pairs"

Section/Topic	Item No.	Recommendation	Reported in manuscript	Notes
**Title and abstract**				
	1a	Indicate the study's design with a commonly used term in the title or the abstract	Title; Abstract (Methods)	Title: "A Prospective Cohort Study"; Abstract: "In this prospective cohort…"
	1b	Provide in the abstract an informative and balanced summary of what was done and what was found	Abstract	Structured abstract (Background/Objectives, Methods, Results, Conclusions)
**Introduction**				
Background/rationale	2	Explain the scientific background and rationale for the investigation being reported	Section 1 (Introduction), paragraphs 1–5	
Objectives	3	State specific objectives, including any prespecified hypotheses	Section 1, final paragraph; Section 2.1	Primary and secondary aims; hypothesis on maternal oral hygiene
**Methods**				
Study design	4	Present key elements of study design early in the paper	Section 2.1	Prospective longitudinal cohort; assessments at T0 (third trimester) and T12
Setting	5	Describe the setting, locations, and relevant dates, including periods of recruitment, exposure, follow-up, and data collection	Sections 2.3, 2.4, 2.5	Setting; recruitment April 2023–May 2024; T0 at gestational weeks 34–38; T12 data collection April 2024–May 2025
Participants	6a	Give the eligibility criteria, and the sources and methods of selection of participants. Describe methods of follow-up	Sections 2.3, 2.5	Eligibility; consecutive recruitment from family medicine practices, assessment at the Faculty of Dental Medicine; at T12 participants were contacted by telephone and completed the questionnaire online
	6b	For matched studies, give matching criteria and number of exposed and unexposed	—	Not applicable (not a matched study)
Variables	7	Clearly define all outcomes, exposures, predictors, potential confounders, and effect modifiers. Give diagnostic criteria, if applicable	Sections 2.4.1–2.4.4, 2.6, 2.7, 2.8	ICDAS and PCR criteria; definitions of favourable behaviours; outcomes; predictors of the main and extended models

Section/Topic	Item No.	Recommendation	Reported in manuscript	Notes
Data sources/ measurement	8	For each variable of interest, give sources of data and details of methods of assessment (measurement). Describe comparability of assessment methods if there is more than one group	Sections 2.4–2.5, 2.9; Supplementary File S1	Clinical examination and questionnaires (full instruments in File S1); intention items mirrored T12 items
Bias	9	Describe any efforts to address potential sources of bias	Sections 2.4.4, 2.6, 2.9; Section 4.9	Examiner calibration (weighted kappa 0.83; ICC 0.89); mirrored items; no intervention after T0; potential biases discussed in Limitations
Study size	10	Explain how the study size was arrived at	Section 2.2	Sample size for the exact McNemar test (Connor method); ≥151 pairs required; 159 analysed
Quantitative variables	11	Explain how quantitative variables were handled in the analyses. If applicable, describe which groupings were chosen and why	Sections 2.4.1–2.4.3, 2.8	ICDAS grouped 0–2 vs. 3–6; PCR continuous (per 10 percentage points), 50% threshold descriptive only; knowledge score 0–6; education in two categories
Statistical methods	12a	Describe all statistical methods, including those used to control for confounding	Sections 2.8, 2.10	Exact McNemar test; multivariable logistic regression with a priori predictors; aOR with 95% CI
	12b	Describe any methods used to examine subgroups and interactions	—	Not applicable (no subgroup or interaction analyses were performed)
	12c	Explain how missing data were addressed	Sections 2.6, 2.7	Coding of “Don’t know”/“I don’t remember” answers; complete-case analysis of pairs with evaluable data in all six domains
	12d	If applicable, explain how loss to follow-up was addressed	Sections 2.3, 3.1, 4.9	Participants lost to follow-up were not included; not formally compared with the analytical sample (acknowledged in Limitations)
	12e	Describe any sensitivity analyses	—	No sensitivity analyses were performed; extended models (Section 2.8) were exploratory
**Results**				
Participants	13a	Report numbers of individuals at each stage of study—eg numbers potentially eligible, examined for eligibility, confirmed eligible, included in the study, completing follow-up, and analysed	Sections 2.3, 3.1	218 enrolled; 177 completed T12; 18 excluded; 159 analysed. Number of women approached and eligible not available

Section/Topic	Item No.	Recommendation	Reported in manuscript	Notes
	13b	Give reasons for non-participation at each stage	Sections 2.3, 3.1	Lost to follow-up (n = 41): did not complete the T12 questionnaire; excluded (n = 18): skipped questions or "I don't remember" answers in at least one domain
	13c	Consider use of a flow diagram	Section 3.1, Figure 1	Participant flow diagram (Figure 1)
Descriptive data	14a	Give characteristics of study participants (eg demographic, clinical, social) and information on exposures and potential confounders	Section 3.1, Table 1	
	14b	Indicate number of participants with missing data for each variable of interest	Sections 2.6, 3.1	Complete-case analysis; 18 participants with skipped questions or "I don't remember" answers in at least one domain were excluded
	14c	Summarise follow-up time (eg, average and total amount)	Sections 2.1, 2.5	Single follow-up at 12 months after birth or shortly thereafter
Outcome data	15	Report numbers of outcome events or summary measures over time	Sections 3.2, 3.3, 3.5; Tables 2–4	Intentions at T0, behaviours at T12 and paired matrices
Main results	16a	Give unadjusted estimates and, if applicable, confounder-adjusted estimates and their precision (eg, 95% confidence interval). Make clear which confounders were adjusted for and why they were included	Section 3.4, Tables 5–6; Section 2.8	Adjusted OR with 95% CI (Tables 5–6); adjustment variables and rationale in Section 2.8. Paired counts in Table 4 allow crude intention–behaviour associations to be derived
	16b	Report category boundaries when continuous variables were categorized	Sections 2.4.1, 2.4.2, 2.8	ICDAS 0–2 vs. 3–6; PCR ≥50% (descriptive); education university vs. non-university
	16c	If relevant, consider translating estimates of relative risk into absolute risk for a meaningful time period	—	Not applicable
Other analyses	17	Report other analyses done—eg analyses of subgroups and interactions, and sensitivity analyses	Sections 3.4 (Table 6), 3.5	Exploratory extended models (ICDAS, PCR); descriptive analysis of night-time breastfeeding
**Discussion**				
Key results	18	Summarise key results with reference to study objectives	Section 4.1	

Section/Topic	Item No.	Recommendation	Reported in manuscript	Notes
Limitations	19	Discuss limitations of the study, taking into account sources of potential bias or imprecision. Discuss both direction and magnitude of any potential bias	Section 4.9	Self-report, social desirability, question–behaviour effect, recall, coding of "Don't know", loss to follow-up, multiplicity, selection bias
Interpretation	20	Give a cautious overall interpretation of results considering objectives, limitations, multiplicity of analyses, results from similar studies, and other relevant evidence	Sections 4.2–4.8; Section 5	
Generalisability	21	Discuss the generalisability (external validity) of the study results	Section 4.9	Single-centre design; high proportion of university-educated participants
**Other information**				
Funding	22	Give the source of funding and the role of the funders for the present study and, if applicable, for the original study on which the present article is based	Funding statement	University of Medicine, Pharmacy, Science and Technology "George Emil Palade" of Târgu Mureș, grant 163/3/10.01.2023; the funder had no role in the design of the study, data collection, analysis or interpretation, writing of the manuscript or the decision to publish

Checklist items reproduced from the STROBE Statement (von Elm E, et al. Lancet 2007;370:1453–1457), available at www.strobe-statement.org. Section numbers refer to the submitted manuscript.
