# Supplementary material for: From Prenatal Intention to Postpartum Practice: A Prospective Cohort Study of Oral Health Preventive Behaviours in Mother–Child Pairs
Source: Healthcare (Basel). 2026 Oct 28;14(21):3652. doi: 10.3390/healthcare14213652 (PMC13762710; doi:10.3390/healthcare14213652)
Supplement: Supplementary file 1 [file healthcare-14-03652-s001.zip › Table_S1_Baseline characteristics of participants included and not included in the final analysis.pdf]

Table S1. Baseline characteristics of participants included and not included in the final analysis.

**Characteristic (T0)**	**Included (n = 159)**	**Not included (n = 59)**	**p**
Maternal age, years, mean ± SD	29.4 ± 4.8	29.8 ± 5.1	0.602
University education, n (%)	96 (60.4)	28 (47.5)	0.087
Primiparous, n (%)	92 (57.9)	33 (55.9)	0.798
Knowledge score (0–6), mean ± SD	4.0 ± 1.3	3.9 ± 1.4	0.634
≥1 tooth with ICDAS 3–6, n (%)	117 (73.6)	42 (71.2)	0.723
PCR, %, mean ± SD	48.0 ± 19.0	50.2 ± 18.5	0.440
Favourable intention – toothbrushing from the first tooth, n (%)	131 (82.4)	47 (79.7)	0.644
Favourable intention – toothbrushing ≥2 times/day, n (%)	132 (83.0)	48 (81.4)	0.774
Favourable intention – fluoride toothpaste, n (%)	108 (67.9)	38 (64.4)	0.624
Favourable intention – no added sugar, n (%)	138 (86.8)	50 (84.7)	0.697
Favourable intention – no night-time bottle, n (%)	104 (65.4)	40 (67.8)	0.741
Favourable intention – first dental visit ≤12 months, n (%)	61 (38.4)	24 (40.7)	0.756

Not included: 41 participants lost to follow-up and 18 excluded because of missing or non-evaluable answers in at least one behavioural domain. SD = standard deviation; ICDAS = International Caries Detection and Assessment System; PCR = Plaque Control Record. p values: independent-samples t-test for continuous variables and Pearson's chi-squared test for categorical variables.
